# Impact of HIV Status on Treatment Outcome of Tuberculosis Patients Registered at Arsi Negele Health Center, Southern Ethiopia: A Six Year Retrospective Study

**DOI:** 10.1371/journal.pone.0153239

**Published:** 2016-04-20

**Authors:** Gebreslassie Gebremariam, Getachew Asmamaw, Muktar Hussen, Mengistu Z. Hailemariam, Demissie Asegu, Ayalew Astatkie, Anteneh G. Amsalu

**Affiliations:** 1 Department of Medical Laboratory Science, Hawassa University, Hawassa, Ethiopia; 2 School of Public and Environmental Health, Hawassa University, Hawassa, Ethiopia; 3 Department of Medical Microbiology, University of Gondar, Gondar, Ethiopia; Hebrew University, ISRAEL

## Abstract

**Background:**

Despite implementation of different strategies, the burden and mortality of human immunodeficiency virus (HIV)-associated tuberculosis (TB) remains a challenge in Ethiopia. The aim of this study was to assess the impact of HIV status on treatment outcome of tuberculosis patients registered at Arsi Negele Health Center, Southern Ethiopia.

**Methods:**

A six-year retrospective data (from September 2008 to August 2014) of tuberculosis patients (*n* = 1649) registered at the directly observed therapy short-course (DOTS) clinic of Arsi Negele Health Center was reviewed. Treatment outcome and tuberculosis type were categorized according to the national tuberculosis control program guideline. Data were entered and analyzed using SPSS version 20. Multinomial logistic regression analysis was used to examine the effect of HIV status separately on default/failure and death in relation to those who were successfully treated. Odds ratios with 95% confidence intervals were used to check the presence and strength of association between TB treatment outcome and HIV status and other independent variables.

**Results:**

Out of the 1649 TB patients, 94.7% (1562) have been tested for HIV of whom 156(10%) were HIV co-infected. The mean (standard deviation) age of the patients was 28.5(15.5) years. The majority were new TB cases (96.7%), male (53.7%), urban (54.7%), and had smear negative pulmonary TB (44.1%). Overall, the treatment success rate of TB patients with or without HIV was 87.3%. Using cure/completion as reference, patients without known HIV status had significantly higher odds of default /failure [aOR, 4.26; 95%CI, 1.684–10.775] and transfer-out [aOR, 2.92; 95%CI, 1.545–5.521] whereas those who tested positive for HIV had a significantly higher odds of death [aOR, 6.72; 95%CI, 3.704–12.202] and transfer-out [aOR, 2.02; 95%CI, 1.111–3.680].

**Conclusion:**

Overall, treatment outcome and HIV testing coverage for TB patients is promising to reach the WHO target in the study area. However, default/failure among patients without known HIV status, and higher rate of mortality among HIV positive TB patients and transfer-out cases deserves concern. Therefore further prospective studies on quality of services, socioeconomics and psychology of this group should be conducted.

## Introduction

The dual tuberculosis (TB) and human immunodeficiency virus (HIV) epidemics are major public health and clinical problems which adversely affect socio-economic development. In tandem, HIV infection and TB create a deadly synergy. TB is the leading cause of death among persons with HIV infection and in areas with a high prevalence of HIV infection, particularly in Sub-Saharan Africa (SSA) [1,2]. According to the recent estimate of the World Health Organization (WHO), there were 9 million new TB cases in 2013 and 1.5 million TB deaths. In the same year (2013) 48% of TB patients globally had a documented HIV test result. This is unacceptable, given that knowledge of HIV status is essential for appropriate treatment. In the African region that has the highest TB/HIV burden; three out of four TB patients knew their HIV status. Of these, 41% tested positive ranging from <12% in Ethiopia, Angola and Mali to 74% in Lesotho and Swaziland[3].

HIV and TB interact synergistically, speeding the progression of illness and increasing the likelihood of death. The presence of HIV enhances the reactivation and progression of latent *Mycobacterium tuberculosis* to overt TB disease, and having TB disease accelerates HIV disease progression[2]. It also alters the clinical presentation of TB and complicates the treatment follow-up. Therefore to reduce the burden of HIV in TB patients, HIV testing and acting upon one’s HIV status is mandatory. For this reason a TB clinic can be an extremely important entry point for HIV prevention, care and treatment [1].

Studies have showed that non-consent for HIV testing is associated with all unfavorable outcomes of tuberculosis treatment[4] and testing positive is associated with lower cure rate, higher death rate[3,4], high failure rate and more default rate [5,6]. Beside socioeconomic and behavioral factors, patient-health care worker poor communication, distance from treatment center and both TB/HIV medications side effects, low CD4 counts, low haemoglobin level, higher viral load, and the presence of other opportunistic infections are predictors of mortality among dually infected patients [7,8]. TB/HIV co-infection is also associated with increasing incidence of smear negative pulmonary TB(SNPTB) and extra pulmonary TB(EPTB) cases[5] and is more difficult for laboratory diagnosis of TB.

Despite strong political commitment and implementation of different strategies such as extensive expansion of directly observed therapy short-course (DOTS) services in Ethiopia[9]and the massive involvement of Health Extension Workers (HEWs) in TB prevention and control activities at the grass-root level, and integrated TB/HIV activities in place[10], the key intervention for reducing the burden and mortality of HIV-associated TB, in particular HIV testing and counseling, enrollment of HIV-positive TB patients on antiretroviral treatment (ART) and provision of cotrimoxazole prophylaxis treatment (CPT) were estimated to be low[3,11]. Since the inception of all the above activities, only few studies have been conducted in Ethiopia[12]. Furthermore, the impact can vary between different population and health systems; hence it is important to evaluate the HIV status and treatment outcomes of TB patients in order to perform interventional activities relying on the gap in specific settings. Therefore, the aim of this study was to assess the impact of HIV status on treatment outcome of TB at Arsi Negele Health Center (ANHC) in Oromia region of Ethiopia.

## Materials and Methods

### Ethical consideration

The study was approved by the Department of Medical Laboratory Sciences ethics committee, College of Medicine and Health Sciences, Hawassa University. Patient records/information was anonymized and de-identified prior to analysis. Permission to collect the information from TB registers was obtained from ANHC administration.

### Study area

Arsi Negele is one of the district towns located in west Arsi zone of Oromia regional state, southern part of Ethiopia. The town is 220 Kilometers south of Addis Ababa, the capital city of Ethiopia. ANHC is one of the public health centers that provide DOTS service for people living in and around Arsi Negele district. Since 2008, the health center also provides provider-initiated voluntary counseling and testing (PICT) of HIV for all TB patients and comprehensive HIV care, treatment and support.

### Study design

A six-year retrospective document review of TB patients registered at the DOTS clinic of ANHC was conducted to assess the impact of HIV on TB treatment outcome.

### Study population

The study population included all TB cases registered from September 2008 to August 2014 at ANHC DOTs clinic. Patients were diagnosed, registered, treated and referred to other DOTs clinics following the national tuberculosis, leprosy and TB/HIV prevention and control program guideline[10].

### Data collection

After identification of TB unit registers, socio-demography (age, sex, and residence), type of tuberculosis infection, history of exposure to anti-tuberculosis treatment, status of HIV infection, and outcome of anti-tuberculosis treatment were collected from April to May 2015 using a checklist prepared for this purpose. Data on ART and provision of CPT for HIV-infected TB patients were not recorded in the TB register and hence information on ART and CPT was not obtained. Patients with incomplete information were excluded.

### Definition and classification of tuberculosis cases

Type of TB and category of TB by previous history of treatment were defined according tothe national TB, leprosy and TB/HIV guideline adopted from WHO[10].

### Treatment outcome

During anti-tuberculosis treatment, smear positive pulmonary tuberculosis (SPPTB) patients are re-examined for acid fast bacilli (AFB) at the end of month 2, 5 and 6 for new cases, and at the end of month 3, 5 and 8 in case of re-treatment. SNPTB and patients with EPTB are monitored clinically and/or radiologically at the same frequency. At the end of the treatment, patients are ranked into mutually exclusive categories as: (a) “**Cured”**–a patient who was initially sputum smear-positive and who was sputum smear-negative in the last month of treatment. If a patient was sputum smear-negative, on at least one previous occasion and sputum smear is not done at the end of treatment, the patient is classified as “**Treatment completed”**. In case of smear negative or extrapulmonary TB, a patient who had received full course of treatment is also referred to as treatment completed. The total of “cured” and “treatment completed” is taken as “**Treatment success**”. (b) “**Treatment failure”–**any TB patient who is initially sputum smear-positive and remained positive at five month or later during treatment. (c) “**Defaulted”**–any patient who had interrupted treatment consecutively for two months or more after taking medication for four weeks and above (d) “**Died”** is a patient who dies from any reason during the course of TB treatment and (e) “Transferred out”—any patient whose treatment results are unknown due to transfer to another health facility[10,13].

### HIV testing and status

The HIV testing in ANHC was performed following the national HIV test algorithm in Ethiopia, where KHB (Shangai Kehua Bio-enginnering Co, Ltd. China) was used for the first screening and positive samples were re-tested with STAT pack (Chembio HIV1/2 STAT pack Assay, USA). Samples giving discordant results in the two tests (KHB and STAT pack) were retested using tie-breaker (Unigold). HIV status was defined as positive, negative and HIV test not done.

### Data analysis

Data were entered, cleaned and analyzed using IBM SPSS version 20 (IBM, USA). Multinomial logistic regression model was used to determine the effect of HIV status on defaulting/failure or death (poor treatment outcome) in contrast to those who were cured/treatment completed (treatment success). Adjusted odds ratios with 95% CIs calculated from the multinomial logistic regression adjusting for possible confounders were used to determine the presence and strength of a statistically significant association between HIV status and TB treatment outcome.

## Results

### Characteristics of patients

Out of 1683 TB patients registered from September 2008 to August 2014 at ANHC, 1649 patients had complete information (98%). Of these, 886 (53.7%) were males while the remaining were females and 902 (54.7%) were urban residents. The mean (SD) age of the patients was 28.5 (15.5) years (range, 2 month to 90 years). Six hundred ninety four (42.1%) patients had SNPTB, 511 (31%) had SPPTB and the rest 444 (26.9%) had EPTB. The majority 1594 (96.7%) of the patients were new TB cases whereas 37 (2.2%) were relapse cases; 8 (0.5%) were treatment failure cases; and 10 (0.6%) were returnees after default. Totally there were 55 (3.3%) re-treatment cases (Table 1).

**Table 1:** Treatment outcome by socio-demographic and clinical profiles of tuberculosis patient at Arsi Negele Health center, 2008–2014.

TB treatment outcomes							
Variables	Total TB cases	Cured N (%)	Completed N (%)	Failure N (%)	Defaulted N (%)	Died N (%)	Transfer N (%)
	1649	424(25.7)	1016(61.6)	7(0.4)	28(1.7)	59(3.6)	115(7.0)
**Age (years)**							
<15	190(6.5)	9(4.7)	167(87.9)	0	6(3.2)	1(0.5)	7(3.7)
15–24	576(31.9)	184(31.9)	326(56.6)	3(0.5)	12(2.1)	8(1.4)	43(7.5)
25–34	410(30.7)	126(30.7)	226(55.1)	3(0.7)	4(1.0)	13(3.2)	38(9.3)
35–44	203(27.1)	55(27.1)	122(60.1)	0	3(1.5)	14(6.9)	9(4.4)
45–54	135(21.5)	29(21.5)	87(64.4)	1(0.7)	2(1.5)	7(5.2)	9(6.7)
55–64	73(19.2)	14(19.2)	45(61.6)	0	0	10(13.7)	4(5.5)
>64	62(11.3)	7(11.3)	43(69.4)	0	1(1.6)	6(9.7)	5(8.1)
**Gender**							
Male	886(53.7)	229(25.8)	525(59.3)	4(0.5)	21(2.4)	40(4.5)	67(7.6)
Female	763(46.3)	195(26)	491(64.4)	3(0.4)	7(0.9)	19(2.5)	48(6.3)
**Residence**							
Urban	902(54.7)	233(25.8)	543(60.2)	3(0.3)	18(2.0)	43(4.8)	62(6.9)
Rural	747(45.3)	191(25.6)	473(63.3)	4(0.5)	10(1.3)	16(2.1)	53(7.1)
**Type of TB**							
SPPTB	511(31.0)	424(83.0)	23(4.5)	6(1.2)	8(1.3)	10(2)	40(7.8)
SNPTB	694(42.1)	0	601(86.6)	1(0.1)	10(1.4)	38(5.5)	44(6.3)
EP TB	444(26.9)	0	392(88.3)	0(0)	10(2.3)	11(2.5)	31(7)
**TB category**							
New	1594(96.7)	394(25.8)	949(62.1)	5(0.3)	28(1.8)	49(3.2)	104(6.8)
Relapse	37(2.2)	19(52.8)	6(16.7)	2(5.8)	0	3(8.3)*	6(16.7)
Failure	8(0.5)	1(14.3)	5(71.4)	0(0)	0	0(0)	1(14.3)
Defaulted	10(0.6)	2(25)	3(37.5)	0(0)	0	3(37.5)	0(0)
**HIV status**							
Not done	87(5.3)	18(20.7)	48(55.2)	1(1.1)	5(5.7)	2(2.3)	13(14.9)
Negative	1406/1562(90)	376(26.7)	884(62.9)	6(0.4)	22(1.6)	31(2.2)	87(6.2)
Positive	156/1562 (10)	30(19.2)	84(53.8)	0(0)	1(0.6)	26(16.7)	15(9.6)

EPTB: Extra-pulmonary tuberculosis; HIV: Human immunodeficiency virus; SNPTB: Smear- negative pulmonary tuberculosis; SPPTB: Smear-positive pulmonary tuberculosis; TB: Tuberculosis; N (%): total number (percentage);

* 2 out of 3 were Multi-drug resistant tuberculosis (MDR TB).

### HIV status

From 1649 TB patients, 1562 (94.7%) were tested for HIV of whom 156 (10% of those tested) were positive and 1406 (90%) were negative. Eighty seven (5.3%) of TB patients didn’t know their HIV status (Table 1).

### Treatment outcomes of TB patients

Overall, the treatment success rate (TSR) of TB patients with or without HIV during the study period was 1440 (87.3%). The remaining 115 (7.0%) were cases of transfer-out, 59 (3.6%) were dead, 28 (1.7%) were defaulters and 7 (0.4%) were cases of treatment failure. Of the HIV positive patients, 30 (19.2%) were cured, 84 (53.8%) were treatment completed, 26 (16.7%) died, 1 (0.6%) was a defaulter, and 15 (9.6%) were transferred-out cases while there was no case of failure. Of the HIV negative TB patients, 376 (26.7%) were cured, 884 (62.9%) were treatment completed, 31 (2.2%) died, 22 (1.6%) were defaulters, 87 (6.2%) were cases of transfer-out and 6 (0.4%) were cases of treatment failure. The TSR for TB among HIV positive cases was 73% while the TSR for HIV negative patients was 89.6% (Table 1).

### Impact of HIV status onTB treatment outcome

In the unadjusted multinomial logistic regression analysis using the combined outcome of cure/ treatment completed (treatment success) as the reference, patients with a positive HIV-status had higher odds of death [COR, 9.27; 95% CI, 5.320–16.153] and transfer-out to other health institutions [COR, 1.91; 95%CI, 1.066–3.405]. On the other hand, patients without known HIV status had higher odds of default/failure [COR, 4.1; 95% CI, 1.637–10.222], and transfer-out [COR, 2.85; 95% CI, 1.515–5.373] compared with the HIV negatives. In the unadjusted analysis, age and type of TB were associated with death; male sex was associated with death and default/failure; retreatment was associated with death and transfer-out and urban residence was associated with transfer-out (Table 2).

**Table 2:** Results of the unadjusted multinomial logistic regression analysis for predictors of the tuberculosis treatment outcome, Arsi Negele Health center, 2008–2014.

	Crude odds ratio (95% confidence interval)			
Variables	Cured/completed*	Default/failure	Deaths	Transfer-out
**Age**, >28 vs. ≤ 28years	1 (reference)	0.55(0.256–1.183)	4.273(2.384–7.660)	0.92(0.617–1.357)
**Sex**, male vs. female	1 (reference)	2.28(1.085–4.770)	1.92(1.099–3.339)	1.27(0.864–1.866)
**Residence**, urban vs. rural	1 (reference)	1.28(0.648–2.544)	2.3(1.283–4.121)	1.0(0.684–1.465)
**Clinical forms**				
SPPTB	1 (reference)	1 (reference)	1 (reference)	1 (reference)
SNPTB	1 (reference)	0.58(0.263–1.299)	2.8(1.393–5.733)	0.82(0.524–1.277)
EPTB	1 (reference)	0.82(0.358–1.854)	1.3(0.527–2.985)	0.88(0.542–1.440)
**Category of TB**,Retreatment vs New	1 (reference)	2.36(0.546–10.230)	7.96(3.736–19.956)	2.53(1.099–5.814)
**HIV serology**				
Not done	1 (reference)	4.1(1.637–10.222)	1.23(0.289–5.257)	2.85(1.515–5.373)
Positive	1 (reference)	0.39(0.053–2.928)	9.27(5.320–16.153)	1.91(1.066–3.405)
Negative	1 (reference)	1 (reference)	1 (reference)	1 (reference)
**HIV test**, not done vs. done	1 (reference)	4.31(1.728–10.733)	0.73(0.175–3.057)	2.65(1.416–4.971)

EPTB: Extra-pulmonary tuberculosis; HIV: Human immunodeficiency virus; SD, standard deviation; SNPTB: Smear-negative pulmonary tuberculosis; SPPTB: Smear-positive pulmonary tuberculosis; TB: Tuberculosis;

*: cured + completed = treatment success

In the multinomial logistic regression analysis adjusting for age, sex, residence, clinical form and category of tuberculosis, the effects of HIV positivity and unknown HIV status on the outcome of treatment were almost similar to those observed in the unadjusted multinomial logistic regression (Table 3).

**Table 3:** Results of the adjusted multinomial regression analysis for predictors of the tuberculosis treatment outcome Arsi Negele Health center, 2008–2014.

	Adjusted odds ratio (95% confidence interval)			
Variable	Cured/completed*	Default/failure	Deaths	Transfer-out
**Age**,>28vs.≤ 28 years	1 (reference)	0.54(0.245–1.172)	2.6(1.411–4.831)	0.85(0.567–1.277)
**Sex**, male vs. female	1 (reference)	2.26(1.070–4.789)	1.59(0.876–2.876)	1.23(0.845–1.847)
**Residence**, urban vs. rural	1 (reference)	1.44(0.719–2.901)	1.74(0.929–3.263)	0.97(0.65–1.431)
**Clinical forms**				
SPPTB	1 (reference)	1 (reference)	1 (reference)	1 (reference)
SNPTB	1 (reference)	0.63(0.280–1.434)	4.04(1.767–9.246)	0.85(0.539–1.342)
EPTB	1 (reference)	0.89(0.381–2.071)	2.7(1.001–7.277)	0.98(0.596–1.626)
**Category of TB**, Retreatment vs New	1 (reference)	2.16(0.469–9.949)	9.5(3.780–24.044)	2.5(1.054–5.935)
**HIV serology**				
Not done	1 (reference)	4.26(1.684–10.775)	1.46(0.335–6.339)	2.92(1.545–5.521)
Positive	1 (reference)	.45(0.059–3.369)	6.72(3.704–12.202)	2.02(1.111–3.680)
Negative	1 (reference)	1 (reference)	1 (reference)	1 (reference)
**HIV test**, not done vs. done	1 (reference)	4.42(1.750–11.161)	0.92(0.215–3.939)	2.72(1.447–5.122)

EPTB: Extra-pulmonary tuberculosis; HIV: Human immunodeficiency virus; SD: Standard deviation; SNPTB: Smear-negative pulmonary tuberculosis; SPPTB: Smear-positive pulmonary tuberculosis; TB: Tuberculosis;

*: cured + completed = treatment success

## Discussion

The failure to provide HIV screening among all TB patients and proper management of TB/HIV co-infected patients leaves the dual epidemics to spread further and become clinically consequential. In this study, a substantial number of TB patients was tested for HIV of whom about 10% were HIV positive. The TSR was worse in tuberculosis patients with HIV than without HIV. We also found that patients without known HIV status had higher odds of default/failure and transfer-out as compared to those tested for HIV. Those who tested positive for HIV had about 7 times higher odds of death and a twofold higher odds of transfer out to other health institutions compared with HIV negative TB patients.

According to the Stop TB plan 2011–2015, 100% of the TB patients should be tested for HIV by 2015[14]. Our study found that 94.7% were tested for HIV. This result is higher than the WHO report for Ethiopia (71%)[3] and than the national TB/HIV sentinel surveillance report 2011-2012 (86%)[15]. The sero-prevalence in the current study was in agreement with a previous study conducted at Enfrazh Health center, northern Ethiopia (11.7%)[16]but lower as compared to several previous studies in Ethiopia in which the sero-prevalence of HIV positivity among TB patients ranges from 18.5% to 67%[12,17–19]. These differences may be attributed to differences in clinical stages of patients visiting health centers and hospitals or due to differences in the prevalence of HIV infection at the community level. Moreover, the declining trend of national HIV infection in the general population from 1.5% in 2011 to 1.1% in 2015 (2014 Spectrum projection) in Ethiopia[20] may explain the differences.

Overall, TSR of registered TB patients with and without HIV was 87.3%consistent with the WHO target of 85%[3].While this result is lower relative to a study at Enfrazh Health Center, northern Ethiopia (94.8%)[16]it is higher as compared to previous findings in Addis Ababa [21], Arsi Zone, central Ethiopia [22], south Ethiopia[23], and northwest Ethiopia[12,19,24].The possible reason for the observed difference might be explained by differences in study setting, high number of transfer out cases[24], high HIV prevalence[12,19,25], increased number of unrecorded treatment outcome in the study in south Ethiopia[23].

The proportion of TB patients without known HIV status were very low (5.3%) as compared to previous studies both in and outside Ethiopia[4,12,23,26]. However, those patients without known HIV status or who tested positive for HIV were more likely to be transferred out to other health institutions as compared to the HIV negatives, and their outcome was unknown. The large number of patients transferred out to other health institutions could adversely impact estimation of TB treatment outcomes, as this group is often included in the denominator. Besides, the feedback system is poor and there are no mechanisms to confirm whether these patients registered to continue treatment in other centers[23]. The possible reason for transfer out of these patients may be due to lack of confidentiality associated with HIV testing or fear of stigma associated with accessing ART. This is an issue in which improvements are needed.

Although studies have shown that patients who default tend to be at higher risk of TB treatment failure (develop MDR-TB) associated with a longer period of TB transmission in the community and had higher rate of mortality[22], still patients from developing countries are interrupting their treatment due to different factors[7]. In this study, those patients without known HIV status had about 4 times higher odds of defaulting and 2.7 times higher odds of transfer-out while testing positive for HIV had no significant association with defaulting or treatment failure. This result partly indicates that knowing HIV status [27] and being HIV positive (probably being on ART, since we have no information about ART usage in the patients in our study)[25,28–31], may serve as protective factors against default from TB treatment. This might be due to psychosocial and economic support for an HIV patient is better than similar support for TB patients (which are very scanty for TB patients in Ethiopia) because of higher number of NGOs and similar organizations involvement initially in HIV care and support. However, in addition to other factors, studies had reported that HIV status[32,33] and being on ART or pill burden[27]were the factors associated with TB treatment non-adherence and lost-to-follow-up.

Unlike a study elsewhere[4], risk of death was not significantly different between HIV negative TB patients and those without known HIV status. However, the risk of death was significantly higher among HIV positive TB patients, concordant with findings from previous studies [4,12,26,34,35]. This finding in some ways may suggest that those patients not tested for HIV are either characteristically similar to those who are HIV negative or may be in an early stages of HIV infection. A number of possible explanations have been proposed for the striking difference in mortality between patients with tuberculosis only and those co-infected with HIV. Immunological studies have shown that the host responses to *M*. *tuberculosis* enhance HIV replication [36], thus accelerating the natural progression of HIV and further depressing cellular immunity. Smear negative pulmonary TB or extra pulmonary TB[4], late presentation and diagnosis of HIV[37], not being on ART[23,28], and the presence of other morbidities like neoplastic diseases[26]in HIV positive TB patients have been implicated in the increased mortality in these patients.

In this study, relative to smear-positive TB patients, the odds of mortality were about 4 times higher among smear-negative patients and 2.7 times higher among extrapulmonary patients. The existing literature [4,5,19]also shows a higher prevalence of HIV in patients with SNPTB and EPTB. The difficulty of TB diagnosis and the consequent delay in treatment initiation may result in higher mortality among SNPTB and EPTB patients[34]. TB diagnosis in Ethiopia has been mainly based on sputum smear microscopy and/or clinically with the decision of clinicians based on chest x-ray result. Currently, there are initiatives to expand Xpert (GeneXpert) MTB/RIF molecular testing to be used for special populations. Yet the implementation is sub-optimal[38] implying that diagnosis is still a challenge.

This study has some limitations which need to be noted while interpreting the findings. As this study was based on a retrospective review of TB registers, comprehensive analysis of all relevant risk factors such as treatment adherence, CD4+ count, opportunistic infections, timing of ART, ART enrollment and provision of CPT do not routinely captured from the record which may overestimate or underestimate the impact of HIV status on TB treatment outcome.

## Conclusions

Overall, treatment outcome and HIV testing coverage for TB patients is promising to reach the WHO target in the study area. However, default/failure among patients without known HIV status, and higher rate of mortality among HIV positive TB patients and transfer-out cases deserves concern. Therefore further prospective studies on quality of services, socioeconomics and psychology of this group should be conducted in order to know the predisposing conditions that may push patients either to abandon the treatment or request for their discharge or transfer to another center.
